# Ubiquitination and De-Ubiquitination in the Synthesis of Cow Milk Fat: Reality and Prospects

**DOI:** 10.3390/molecules29174093

**Published:** 2024-08-29

**Authors:** Rui Gao, Yanni Wu, Yuhao Wang, Zhangping Yang, Yongjiang Mao, Yi Yang, Chunhua Yang, Zhi Chen

**Affiliations:** 1 College of Animal Science and Technology, Yangzhou University, Yangzhou 225009, China; mx120230881@stu.yzu.edu.cn (R.G.);; 2 Institute of Biological Resources, Jiangxi Academy of Sciences, Nanchang 330029, China

**Keywords:** ubiquitination, de-ubiquitination, proteasome, fatty acids, milk fat

## Abstract

Ubiquitination modifications permit the degradation of labelled target proteins with the assistance of proteasomes and lysosomes, which is the main protein degradation pathway in eukaryotic cells. Polyubiquitination modifications of proteins can also affect their functions. De-ubiquitinating enzymes reverse the process of ubiquitination via cleavage of the ubiquitin molecule, which is known as a de-ubiquitination. It was demonstrated that ubiquitination and de-ubiquitination play key regulatory roles in fatty acid transport, de novo synthesis, and desaturation in dairy mammary epithelial cells. In addition, natural plant extracts, such as stigmasterol, promote milk fat synthesis in epithelial cells via the ubiquitination pathway. This paper reviews the current research on ubiquitination and de-ubiquitination in dairy milk fat production, with a view to providing a reference for subsequent research on milk fat and exploring new directions for the improvement of milk quality.

## 1. Introduction

Milk is known as a “whole food” that meets the nutritional needs of human beings due to its rich nutrient content. Milk fat not only is the main energy component of milk but also determines the physical properties, manufacturing characteristics, and organoleptic qualities of dairy products [1,2]. Increasing the nutrient content of milk improves the health of infants and young children. Higher fat rates provide cost savings and increased revenue for the dairy industry [3]. Therefore, determining how to improve milk fat percentage has been an urgent scientific problem in the international dairy industry. The study of the milk fat synthesis network from the perspective of molecular biology has become a research hotspot in this field.

Ubiquitin, a small-molecule protein consisting of 76 amino acid residues, is widely found in a variety of eukaryotic organisms. It was first identified from reticulocytes and is called APF-1, and it is a key factor in the ATP-dependent hydrolysis of substrate proteins [4]. Remarkably, ubiquitin proteins are among the most conserved proteins known. There are only three different amino acid residues between ubiquitin in yeast and humans, which makes ubiquitin functionally interchangeable and universal across species [5,6]. Ubiquitin primarily labels proteins that need to be degraded and then mediates the degradation of target proteins via the ubiquitination–proteasome and lysosomal pathways. Diverse ubiquitin chains influence the biological function of target proteins. For example, the activity of certain transcription factors can be enhanced by self-ubiquitination or by interaction with specific ubiquitin ligases [7,8]. The de-ubiquitination of proteins refers to reversing the ubiquitination process by the operation of de-ubiquitinating enzymes (DUBs) [9]. Ubiquitination and de-ubiquitination mediate approximately 80–85% of the protein degradation in eukaryotic cells [10]. Together, they maintain the homeostasis of the intracellular environment and the integrity of organelles and play a crucial role in protein location, metabolism, function, regulation, and degradation [11].

It has been demonstrated that ubiquitination and de-ubiquitination can regulate the expression of components related to milk fat synthesis in cattle. Both high-glucose concentrations and treatment with proteasome inhibitors have been reported to increase the synthesis of triglycerides (TAGs) in bovine mammary epithelial cells, accompanied by a marked accumulation of ubiquitin proteins [12]. Some de-ubiquitinating enzymes can indirectly increase the activity of fatty acid synthase (FASN) and peroxisome proliferator-activated receptor y (PPARy) to promote fatty acid synthesis from scratch [13]. Abnormal regulation of the ubiquitination pathway can block the ubiquitination degradation of fatty acid transporter proteins in the cell membrane, resulting in enhanced fatty acid transport. In addition, the ubiquitination of some components can enhance their own function and benefit more fatty acid synthesis [14].

With the increasing number of components related to the ubiquitination and de-ubiquitination of milk fat synthesis being unearthed, this may herald the existence of a complex ubiquitin-led regulatory network in milk fat synthesis in dairy cows. Investigating the biological functions and mechanisms of ubiquitination and de-ubiquitination in milk fat production in dairy cows may provide a new theoretical basis for exploring the molecular mechanism of milk production traits and molecular breeding in dairy cows. This paper reviews the current research on the ubiquitination and de-ubiquitination pathways in milk fat production in cattle and draws on related studies on mammals with the purpose of laying a foundation and discovering new directions for later studies on milk fat in cows.

## 2. Ubiquitination and De-Ubiquitination Systems

### 2.1. Ubiquitinase and Ubiquitination Reactions

Ubiquitination is a post-translational modification process in which ubiquitin is covalently bound to target proteins through the combined action of ubiquitin-activating enzyme (E1), ubiquitin-conjugating enzyme (E2), and ubiquitin protein ligase (E3), mainly regulating the stability of endogenous proteins [15,16,17]. E1 is a promoter of ubiquitination modification, which binds ubiquitin by adenylating the -COOH terminus of ubiquitin with the aid of Mg catalysis and the hydrolysis of ATP, followed by the formation of a high-energy thioester bond between a cysteine (Cys) in its own active site and the ubiquitin C-terminal Gly 76. [18,19]. E2 is present in almost all eukaryotes and acts as a ubiquitin transfer platform, where E1 transfers activated ubiquitin to cysteine residues of E2, forming the E2–ubiquitin thioester complex by ester exchange [20,21,22]. Ultimately, E3 recruits and binds the E2–ubiquitin thioester complex and the substrate, catalysing the formation of an isopeptide bond between the C-terminal carboxyl group of ubiquitin and either the lysine side chain or the free N-terminal amino group of the substrate, which results in the transfer of ubiquitin from E2 to the target protein [23,24]. Ubiquitin ligase E3 has three family members, a really interesting new gene (RING), homologous to the E6AP carboxyl terminus (HECT), and a RING-in-between-RING (RBR) [25]. The RING mediates the catalytic transfer of ubiquitin directly from E2 to the substrate, while the remaining two catalyse the reception of ubiquitin using their own cysteine residues and then transfer ubiquitin to the target proteins. The unusually high abundance of genes coding for E3 allows for precise substrate selection [26,27]. Of course, ubiquitinating enzymes can themselves be modified by ubiquitination.

### 2.2. Ubiquitin–Substrate Linkages

**Figure 1:**
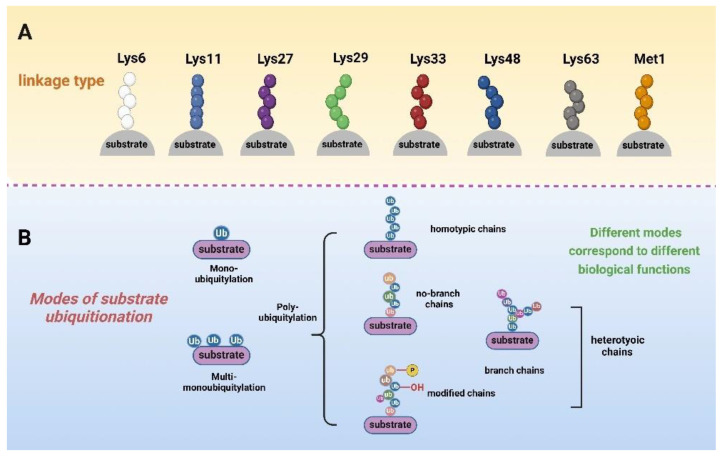
Schematic representation of how ubiquitin is linked to substrates, with differently coloured spheres representing ubiquitin molecules linked by different sites. (**A**) Both Met1 and the seven Lys residues in ubiquitin can form specific chain bonds with different conformations. (**B**) Substrates can be modified by mono-, multi-mono-, or polyubiquitin. Polyubiquitin includes homo- and hetero-chains. Heterodimeric chains include both homo- and heterodimeric chains, and heterodimeric chains have branched forms in addition to straight chains. In addition, ubiquitin is affected by other post-translational modifications, such as phosphorylation and hydroxylation.

The ubiquitin molecule contains seven lysine sites, including Lys-6, Lys-11, Lys-27, Lys-29, Lys-33, Lys-48, Lys-63 (K6, K11, K27, K29, K33, K48, and K63), and the N-terminal Met1 residue, all of which are involved in ubiquitin chain formation (Figure 1A). The ubiquitin bonds in ubiquitin and these residues can form specific chain bonds with different conformations and specific (or unknown) functions. In addition, the substrate can be modified by one or more ubiquitination. According to the way ubiquitin molecules link to substrates, they can be roughly divided into three types of modifications [28,29]. The first type is mono-ubiquitylation, where only one ubiquitin molecule binds to the substrate [30]. The second is multi-monoubiquitylation, in which multiple ubiquitin molecules do not touch each other and bind to different sites on the substrate [31]. The last is polyubiquitylation, where the first bound ubiquitin can act as a receptor for the later bound ubiquitin [32]. The steady-state level of ubiquitin is the result of a dynamic equilibrium between these forms [33]. Multiple ubiquitin molecules can form polyubiquitin chains, both linear and branched, through the different residues mentioned above, and then go on to modify the substrate [34]. Modifications of the same or different residues lead to the formation of homogeneous or heterogeneous chains, respectively [35]. More importantly, the ubiquitin fraction can also be modified by other post-translational modification pathways (Figure 1B). For example, Ser65 phosphorylation and Ser57 phosphorylation are the most widely studied phosphorylation events on ubiquitin. Different linkages and modifications fulfil diverse biological functions [36]. For instance, polyubiquitination at the K48 site is involved in the selective degradation of aberrant proteins, cell cycle regulation, and immune response [37,38]. K63 chains can be involved in protein transport and DNA damage repair and play an important role in the NF-κB signalling pathway [39].

### 2.3. Ubiquitin-26S Proteasome System (UPS)

**Figure 2:**
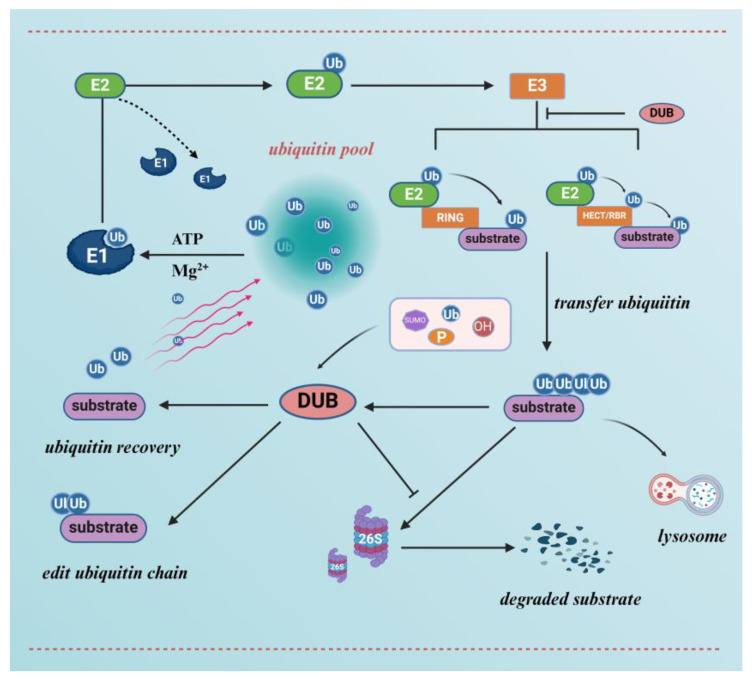
A functional model of ubiquitin-mediated degradation of substrate proteins and de-ubiquitinating enzymes. The ubiquitin molecule undergoes labelling of the substrate protein by ubiquitin-activating enzyme E1, ubiquitin-conjugating enzyme E2, and ubiquitin–lyase E3, followed by degradation of the substrate protein via the 26S proteasome or lysosome. De-ubiquitinating enzymes (DUBs) can inhibit the action of ubiquitin ligases to antagonise the ubiquitinated degradation of substrates, as well as edit ubiquitin chains by cleaving ubiquitin, facilitating the recovery of ubiquitin molecules. In addition, de-ubiquitinating enzymes are regulated by various post-translational modifications.

The 26S proteasome is a large multisubunit complex found in eukaryotes and prokaryotes. Substrates labelled with ubiquitin are subsequently degraded by the 26S proteasome or perform non-protein hydrolysis functions, releasing individual amino acid residues that can be used for new protein synthesis [40]. The 26S proteasome can be structurally divided into two parts: the 19S regulatory granule and the 20S core granule [41,42,43]. The 19S regulatory particle is responsible for recognising and unfolding the ubiquitin-chain-labelled protein substrate, which is then transported to the 20S core particle for degradation. 20S core particles are associated with caspase and trypsin activity. Finally, the substrate is digested into a peptide of 2–24 amino acids [41,43,44] (Figure 2). Furthermore, there is a coordinated and complementary relationship between UPS and the cell’s autophagic system [45]. Certain ubiquitinated transporter membrane proteins can be transported for lysosomal degradation mediated by protein-sorting transporter devices (ESCRTs), which benefit from a labelling system that co-uses ubiquitination as a substrate [46,47]. The autophagic lysosomal pathway is an important compensatory mechanism mediating the degradation of ubiquitinated protein aggregates [48,49].

### 2.4. De-Ubiquitinating Enzymes (DUBs) and De-Ubiquitinating Systems

Ubiquitination can be reversed by DUBs, which counteract ubiquitin ligase activity by binding to ubiquitin-based isopeptide bonds, thereby cleaving ubiquitin from the substrate protein, in addition to cleaving ubiquitin molecules in the lower ubiquitin chain [50,51]. DUBs usually contain one or more binding structural domains for substrate recognition and are based on the composition of their catalytic structural domains. DUBS can be classified into six major families. Most DUBs are cysteine proteases. The USP family is the most abundant, with more than fifty members involved in a variety of biological functions, including cell proliferation, differentiation, apoptosis, and migration [52,53,54]. DUBs also have the ability to antagonise E3 auto-ubiquitination, for example, in the endoplasmic reticulum, where the multi-spanning ubiquitin ligase Hrd1 is responsible for the formation of reverse transcription translocation channels, and Hrd1 auto-ubiquitination is counteracted by the de-ubiquitinating enzyme Ubp1, thus maintaining the stability of endoplasmic-reticulum-associated protein degradation processes [55]. In turn, DUBs are targets of E3-mediated ubiquitination, and it has been demonstrated that the two cooperate to determine protein ubiquitination [56,57]. For example, DUBs can restrict or regulate the key types of polyubiquitin generated by E3 [58,59]. In addition, ubiquitinating enzymes undergoing self-ubiquitination can be rescued by DUBs. Indeed, multiple post-translational modifications, including ubiquitination, can regulate DUBs (Figure 2). It has been revealed that in order to participate in cellular signalling pathways, PTM can regulate the function of DUBs by altering their stability, localisation, abundance and catalytic activity [60].

## 3. Ubiquitination and De-Ubiquitination Modifications Are Closely Related to Milk Fat Synthesis in Dairy Cows

The main component of milk fat is triglyceride, which accounts for 95~98% of the total milk fat. Milk fat globules (MFGs), which are wrapped in a phospholipid membrane, are the only form of milk fat. In addition, milk fat also contains small amounts of phospholipids, cholesterol, and free fatty acids, which are further divided into saturated and unsaturated fatty acids [61,62]. The biology of lactation in dairy cows has established that fatty acids and glycerol for the synthesis of triglycerides in milk fat originate from two main pathways: absorption from the blood and self-synthesis in mammary epithelial cells [63]. However, both directly absorbed fatty acids and short- and medium-chain fatty acids synthesised from scratch need to be transported, activated, lengthened, and desaturated by a series of enzymes in the cell before triglycerides can finally be synthesised. Thus, the process of milk lipid production requires precise regulation by a series of receptor–ligand binding, transport carriers, and enzymes, while the activation and degradation of these molecules require the dynamic regulation of ubiquitination and de-ubiquitination.

### 3.1. Overview of Milk Fat Synthesis

Long-chain fatty acids (LCFAs) in the mammary gland cannot be synthesised from scratch. They can only be absorbed actively or passively from the circulating blood, which, of course, cannot be carried out without the work of fatty acid transporter membrane proteins [64]. Briefly, free fatty acids are released from very low-density lipoproteins (VLDLs) and celiac microparticles (CMs) in the blood in cooperation with lipoprotein lipase (LPL) and very low-density lipoprotein receptors (VLDLRs). Some short-chain fatty acids (SCFAs) can enter the mammary epithelial cells via passive transport, while LCFAs are mainly transported by fatty acid transporter proteins (CD36). After passing through the membrane, LCFAs are activated by recombinant acyl coenzyme A synthetase (ACSL) to form LCFA-CoA, which is subsequently transported to the endoplasmic reticulum by fatty acid binding proteins (FABPs) [65]. During fatty acid transport, CD36, ACSL, and FABPs play crucial roles in regulating long-chain fatty acid transport and intracellular long-chain fatty acid concentration.

In contrast, the process of SCFA de novo synthesis is very complex and requires a variety of enzymes to catalyse the reaction. Roughly speaking, carbohydrates from the diet enter the rumen of the cow, where they are fermented by rumen bacteria to produce acetic acid and butyric acid, which are then oxidised to β-hydroxybutyric acid (BHBA). Next, they are transported through the blood circulation and passively diffuse through the endothelial and mesenchymal spaces of the capillaries into the mammary epithelial cells [66]. After activation of acyl-coenzyme A synthetase (ACSS), important precursors, such as acetic acid and β-hydroxybutyric acid, are catalysed by acetyl coenzyme A carboxylase (ACACA) and fatty acid synthase (FASN) for de novo synthesis of the SCFAs. Eventually, FA-CoA, with different numbers of C atoms, is formed [67,68]. Various short and long FA-CoAs are transported by FABPs to the endoplasmic reticulum in order to be desaturated by stearoyl coenzyme A desaturase 1 (SCD1) and, finally, to generate TAG via the 3-phosphoglycerol pathway [69,70,71,72].

**Figure 3:**
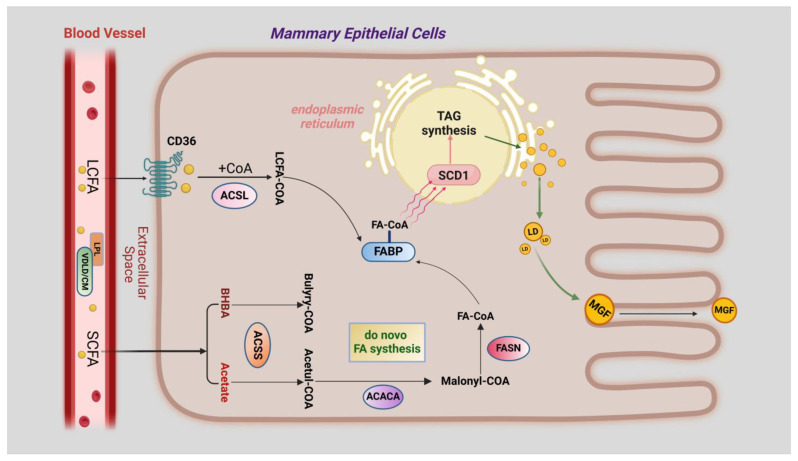
A model of the milk lipid synthesis process in the mammary epithelial cells of dairy cows. LCFA is translocated into the cell via CD36 and forms LCFA-CoA in the presence of ACSL. SCFAs can be taken up directly by the cell and form FA-CoA catalysed by ACSS, ACACA, and FASN. These acyl-coenzyme A molecules are translocated into the endoplasmic reticulum via FABPs and undergo desaturation and triglyceride formation.

Triglycerides are formed as droplets in the endoplasmic reticulum of mammary epithelial cells. Adipose differentiation-related protein (ADFP) is located on the surface of intracellular lipid droplets (LDs) and plays a key role in facilitating the production of lipid droplets. These lipid droplets aggregate between the bilayers of the endoplasmic reticulum and then wrap around a membrane derived from the endoplasmic reticulum and are released into the cytoplasm as outgrowths. Small lipid droplets fuse with each other to form large milk fat globules and move towards the apical part of the cell, where they are eventually released into the lumen of the mammary alveoli [73]. The process of milk fat generation described above is presented in the form of a picture in Figure 3.

### 3.2. Ubiquitination and De-Ubiquitination during Fatty Acid Transport

CD36 is a transmembrane glycoprotein located on the cell membrane and plays a major role in LCFA uptake and transport [74]. Yang et al. found a significant reduction in the level of cellular fat synthesis after blocking CD36 protein in the mammary epithelial cells of dairy cows [75]. Increasing the level of CD36 proteins in rat skeletal muscle cells by means of electro-transfection contributed to the fatty acid transport efficiency [76]. CD36 is also involved in intracellular signal transduction events that affect LCFA utilisation and metabolism in a variety of cells [77,78].

It was found that the fatty acid content in C2C12 myofibroblasts significantly increased polyubiquitination at the Lys 469 and 472 sites in CD36 [79]. Zheng et al. reported a significant decrease in the level of ubiquitination of CD36 in human foam cells in response to treatment with the adipocytokine CTRP9, which is highly homologous to lipocalin, protecting CD36 from degradation by the proteasome and thus promoting the efflux of excess lipids [80]. These studies all indicate that CD36 is indeed directly subject to ubiquitination modification. In addition, in dairy mammary epithelial cells, Liu et al. found that the knockdown of VPS28, a subunit of endosomal sorting complexes required for transport (ESCRTs), resulted in increased ubiquitination of the CD36 protein, decreased lysosomal degradation of CD36, and was accompanied by more long-chain fatty acid transport into the cytosol. At the same time, DUBs were used to hydrolyse ubiquitin molecules on membrane proteins for their recycling [81]. The de-ubiquitination of CD36 has not been reported in cows; however, Zhang et al. found that in murine macrophages, the de-ubiquitinating enzyme USP14 reduced the anchoring time of ubiquitin-coupled compounds and stabilised CD36 by inducing de-ubiquitination of the CD36 protein [82]. The de-ubiquitinating enzyme UCHL1 promoted the de-ubiquitination of CD36 in human macrophages, reducing the abundance of K48-polyubiquitin on CD36 and avoiding macrophage-to-foam cell transition [83]. These living cases demonstrate that CD36 can be directly regulated by ubiquitination or de-ubiquitination and primarily affects the transmembrane transport of fatty acids [84].

The inhibition of proteasome activity in the mammary epithelial cells of dairy cows resulted in increased mRNA abundance in the *ACSL1* and *FABP3* genes and a significant increase in LCFA content, suggesting that they are indeed subject to ubiquitin–proteasomal regulation, and thus, affect fatty acid transport [81].

The ACSL family is mainly located on the outer mitochondrial membrane and is responsible for the acylation of LCFAs that are transported into the cell into LCFA-CoA [85,86]. Bionaz et al. investigated the changes in the expression of the ACSL family in dairy cows during pre-lactation and lactation and found that the expression of the *ACSL* gene was significantly elevated at the peak of lactation, promoting the formation of milk fat [86]. ACSL1 isoforms predominate in bovine mammary tissues. Unfortunately, direct ubiquitination and de-ubiquitination studies of ACSL1 proteins have not yet been reported. However, it has recently been demonstrated that YAP promotes NEDD4L-mediated ubiquitination of ACSL4 (another member of the ACSL family, similar in structure to ACSL1) [87]. The involvement of YAP as a major progenitor of the hippo signalling pathway in the regulation of milk fat synthesis is well established [88]. A good question is whether YAP would then mediate ubiquitination or de-ubiquitination of the ACSL family in the mammary epithelial cell.

FABPs are a family of fatty acid-binding proteins involved in the transport and utilisation of acylated fatty acids in mammary epithelial cells [89]. For example, CD36 and FABP3 are expressed simultaneously and synergistically in mammary epithelial cells, and FABP5 regulates SREBP-1c protein expression to promote milk fat synthesis [90]. Chung et al. found that FABP3 is a target for the regulation of ubiquitination-like SUMOylation [91]. According to a recent report, in human hepatocytes, the ubiquitin ligase TRIM45 directly adds K33-type and K63-type polyubiquitin chains to the NLS structural domain of FABP5, which promotes the nuclear translocation of FABP5, and the activated downstream pathway promotes triglyceride synthesis [92]. Derlin-1 (an E3 ubiquitin ligase regulator) promotes the ubiquitination of FABP1 and thus regulates lipid accumulation [93]. This suggests that the FABP family is amenable to ubiquitination modifications, but of course, the potential for de-ubiquitination remains to be explored.

### 3.3. Ubiquitination and De-Ubiquitination in the De Novo Synthesis of Fatty Acids

Sterol regulatory element binding proteins (SREBPs) are a class of proteins that play a regulatory role in the metabolism of fatty acids and sterols [94,95]. By resequencing the coding and conserved non-coding regions of the SREBP gene in 423 Holstein cows from five populations, Rincon et al. found that six SNPs were associated with milk fat traits [96]. Li et al. showed that the silencing of SREBP1 in bovine mammary epithelial cells decreased the expression of genes related to lipid synthesis and reduced triglyceride secretion [97].

The approximate process by which SREBP functions is as follows: a protein-cleavage-activating protein (SCAP) first binds to SREBP and moves towards the endoplasmic reticulum, followed by reversible binding to insulin-inducible genes (INSIG) to form the SREBP-SCAP-INSIG complex. In the absence of steroids in the cell, the binding of SREBP-SCAP to INSIG is disrupted, and the SCAP transports SREBP to the Golgi. In the Golgi, successively cleaved by the protein hydrolases S1P and S2P, the SREBP precursor protein releases the N-terminal transcriptional activation region, the mature form of SREBP (nSREBP) [98,99]. Mature SREBP transfers to the nucleus and binds to the sterol-binding element SRE on the target gene to activate transcription. For example, in the mammary gland of goats, SREBP-1 regulates transcription of *ACSS2* by binding to the SRE element of the *ACSS2* promoter [100]. Indeed, the stable presence of nSREBP protein directly affects the expression level of target genes [101,102].

Earlier, it was proposed that mature active SREBP is at least partially degraded by ubiquitination, but the exact mechanism was not clear. It was not until Anders Sundqvist published a study in which the behaviour of SREBP binding to target genes subsequently drove SREBP’s own phosphorylation and ubiquitination processes. This is a specific response mechanism; specifically, when SREBP1 binds to a target gene, in response to this DNA binding, SREBP1 recruits GSK-3β, allowing SREBP1 to undergo phosphorylation, and phosphorylated SREBP1 recruits FBW7 (the substrate recognition protein of the SCF-type ubiquitin ligase complex) to bind to its own promoter region. Labelling FBW7 ultimately leads to the ubiquitination modification of SREBP1 and its degradation via the proteasome pathway [103]. Different SREBP isoforms may have different GSK-3β-mediated phosphorylation. For example, SREBP1 is phosphorylated at the Thr426 and Ser430 sites [104]. Apparently, the loss of SREBP1 activation decreased the expression activity of downstream genes, such as *ACCS*, *FASN*, and *SCD1*, thus reducing the de novo synthesis of fatty acids.

During fat synthesis, the de-ubiquitination of SREBPs has rarely been reported. SREPB2 is more involved in cholesterol synthesis [105]. Although milk fat contains only a small amount of cholesterol, it is also an indispensable nutrient for the human body. The mammalian de-ubiquitinating enzyme USP28 is shown to control the stability of many proteins critical for physiological activity [106]. For instance, USP28 repairs DNA damage in cells by de-ubiquitinating stabilising checkpoint kinase 2 (CHK2) and TP53-binding protein 1 (TP53BP1) [107,108]. A recent study found that USP28 can de-ubiquitinate ubiquitin-tagged SREBP2 to antagonise FBW7-mediated ubiquitination, maintaining the stability of the SREBP2 protein and ensuring essential cholesterol synthesis [109].

For SREBP activation, it was found that trans-10, cis-12 conjugated linoleic acid inhibits the binding of ubiquitin-like domain-containing protein 8 and Insig1 and reduces the proteasomal degradation of Insig1, thereby playing a role in the activation of SREBP1 [110]. As mentioned above, SREBP needs to be escorted by the SCAP to reach the Golgi. The mutual separation of luminal loops 1 and 7 of the SCAP in the transient presence of the endoplasmic reticulum leads to the concealment of ER export signals. RNF5 (an endoplasmic reticulum-anchored E3 ubiquitin ligase), which is widespread and species-conserved on the endoplasmic reticulum, can mediate the polyubiquitylation of the SCAP’s Lys-305 locus and Lys-29, which ultimately strengthens the interaction of luminal loops 1 and 7. This ubiquitination modification ensures the normal transport of SREBP and lays the foundation for the formation of a more mature SREBP, which also implies the foundation for fatty acid and cholesterol synthesis [111].

FASN is a complex homomeric enzyme that plays a central role in mammalian de novo fat production by cyclic extension of activated precursors through two carbon units to form a 16-carbon-atom fatty acid (a conserved chemical reaction) [112]. It was shown that fatty acid and triglyceride synthesis was significantly increased by enhancing the activity of the *FASN* promoter in the mammary epithelial cells of cows [113]. Polymorphisms in the *FASN* gene contribute to changes in the milk fat content as cows enter lactation while supporting the maintenance of mammary gland development and lactation functions [114,115]. The tripartite motif (TRIM) family of proteins comprises a large class of E3 ubiquitin ligases containing the RING structural domain and, excitingly, is a conserved family of proteins [116]. In human hepatocytes, a recent study screened for the lipid negative regulator TRIM56 and conclusively determined that TRIM56 induces degradation of the proteasomal pathway of FASN through catalytic K48-type ubiquitination, thereby inhibiting FASN protein expression and downstream lipid synthesis reactions [117]. Also in hepatocytes, Zhang et al. found that TRIM21 also promotes FASN degradation through K48-type ubiquitination. These results may imply that the ubiquitination mode of the K48 chain has a strong specificity to follow FASN [118]. In contrast, de-ubiquitination studies of FASN have been commonplace. Members of the USP family of de-ubiquitinating enzymes in mammals have been reported to negatively regulate proteasome activity by disassembling ubiquitin chains as well as through non-catalytic mechanisms [119,120]. For example, in mouse hepatocytes, USP14 can enhance the stability of FASN by blocking its ubiquitination and degradation to regulate TAG homeostasis in the liver [121]. In mouse adipocytes, deletion of the de-ubiquitinating enzyme USP19 results in decreased FASN expression, accompanied by reduced synthesis of triglycerides [122]. In addition, the level of ubiquitination in mammary epithelial cells alters the expression of FASN, which makes us wonder whether the ubiquitination of FASN in cow mammary epithelial cells is not likely to involve the K48 chain as well, and whether the USP family is the most probable de-ubiquitinating enzyme for it. Of course, these are all things we need to verify in the future.

### 3.4. Ubiquitination and De-Ubiquitination in the Desaturation of Fatty Acids and Lipid Droplet Formation

The unsaturated fatty acids in milk have been shown to have many benefits, such as contributing to lipid metabolism in the blood, circulation, and antioxidants in the skin [123]. As the standard of living rises, people are increasingly concerned about the content of unsaturated fatty acids in milk. SCD1 is a key enzyme in the biosynthesis of monounsaturated fatty acids in the mammalian endoplasmic reticulum and catalyses the synthesis of long-chain fatty acids with a double bond in the cis-Δ9 position [124]. The expression of mammary SCD1 increases dramatically during lactation in cows. In mammalian cells, the inhibition of proteasome activity leads to the accumulation of ubiquitinated SCD1, and it was determined with immunoprecipitation that SCD1 is modified by polyubiquitination [125].

In mammary cells, PPARy can act directly on SCD1 and upregulate the expression of SCD1, corresponding to decreases in the levels of C16:0 and C18:0 and increases in the levels of C16:1 and C18:1 [126]. Several reports have shown that PPARy is degraded by the ubiquitin–proteasome system and that, structurally, the ligand-binding structural domain (LBD) of PPARy contains the major ubiquitination site [127,128]. FBXO9 ubiquitin ligase was recently identified in adipocytes, and the overexpression of FBXO9 resulted in reduced endogenous PPARy levels and inhibited adipogenesis [129]. In human hepatocytes, the de-ubiquitinating enzyme USP22 promotes lipid synthesis by de-ubiquitinating the K48 chain of PPARy [130]. Unfortunately, the exact ubiquitin ligase has not been identified in bovine mammary cells, although it has been shown that the level of intracellular ubiquitination affects the level of PPARy expression.

ADFP is not only a specific marker of lipid accumulation but also promotes the binding of fat droplets to receptors on the cell membrane. Finally, it releases fat droplets outside the cell through the plasma membrane to form milk fat [131]. ADFP was significantly expressed in the mammary tissue of both adult and lactating dairy cows [132]. Recently, it was found that the ubiquitin ligase Mul1 recognises ADFP and regulates its ubiquitination for degradation [133]. Furthermore, in dairy mammary epithelial cells, the stability of ESCRTs was affected by interfering with the expression of VPS28, and ADFP showed significant accumulation in the cells, suggesting that ADFP is regulated by the ubiquitin–lysosomal system [134].

**Figure 4:**
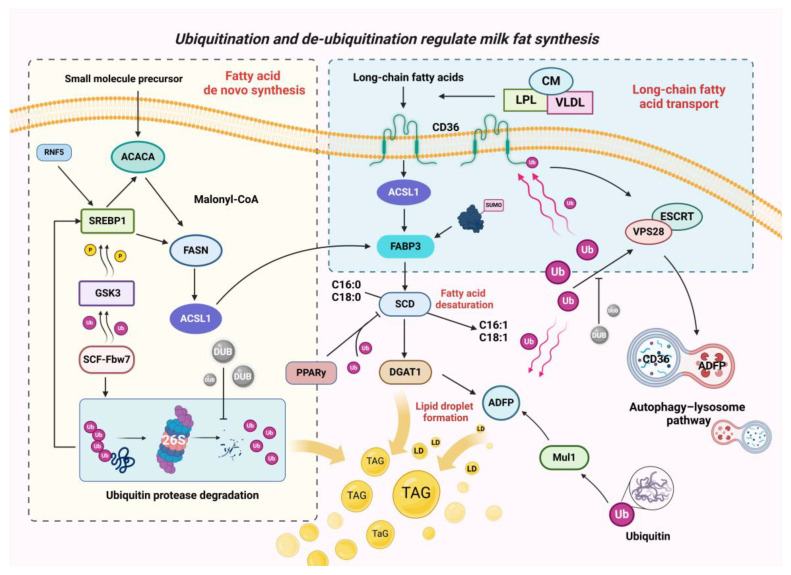
A model of ubiquitination- and de-ubiquitination-regulating components related to milk fat synthesis. In the use of long-chain lipids, ubiquitination regulates the translocation of long-chain fatty acids through the lysosomal pathway and influences the intracellular transport of long-chain fatty acids through ubiquitination-like SUMO chemistry. During fatty acid de novo synthesis, ubiquitination affects fatty acid chain elongation and translocation in cells by regulating SREBP abundance, while de-ubiquitinating enzymes antagonise ubiquitination-mediated degradation of target proteins. In fatty acid desaturation and lipid droplet generation, ubiquitination affects unsaturated fatty acids and TAG formation by influencing PPARy and ADFP.

The regulation of fatty acid transport, de novo synthesis, desaturation, and lipid droplet synthesis through the above ubiquitination and de-ubiquitination modifications is shown in Figure 4.

## 4. Natural Plant Extracts Regulate Milk Fat Synthesis by Ubiquitination Modification

The scientific and rational formulation of feed ingredients ensures that cows can consume sufficient protein, vitamins, minerals, and other nutrients, which improves milk quality [135,136]. Nowadays, more and more attention is being paid to the choice of feed additives for dairy cows, especially new functional feed additives that promote health and environmental sustainability. For example, extracts from natural plant sources, which may have anti-inflammatory or antioxidant properties, have also been shown to contribute to milk fat synthesis [137,138]. However, cows, as ruminants, have a very complex digestive system, and little is known about how these natural plant extracts travel through their various organs or about the molecular mechanisms by which they work. However, focusing on cow mammary epithelial cells, some recent studies have found that they can promote milk fat production, and it is exciting to observe the involvement of ubiquitination modifications.

**Figure 5:**
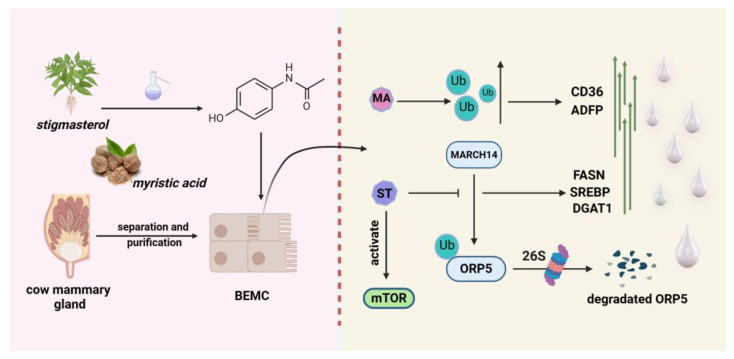
A model for the promotion of milk fat synthesis in dairy cow mammary cells through the ubiquitination pathway by soysterols and myristic acid. Soysterols promote CD36 and ADFP expression by increasing intracellular ubiquitination levels. Myristic acid promotes fatty-acid-synthesis-related gene expression by antagonising the MARCH14-mediated ubiquitination degradation of ORP5. In addition, myristic acid activated the mTOR signalling pathway to assist milk synthesis.

Stigmasterol (ST) is a biologically active phytosterol found in a variety of food sources, such as vegetables, nuts, grains, and potatoes, and is often referred to as the “key to life” [139]. Stigmasterol has a variety of physiological functions, such as lowering cholesterol content, antioxidants, growth promotion, immunomodulation, and so forth [140]. Oxysterol-binding protein-related protein 5 (ORP5) is a member of the OSBP family, which possesses a conserved ORD structural domain and is an essential class of sterol sensors [141]. ORP5 acts as a lipid transfer protein capable of sensing, binding, and transporting lipids between intracellular membranes [142,143]. In a newly published study, they found that in dairy mammary epithelial cells, ST inhibits the ubiquitination of ORP5 proteins caused by the ubiquitin ligase MARCH4, sparing them from proteasomal degradation. Stabilised ORP5 increased the levels of DGAT1, DGAT2, FASN, and SREBP protein expression and promoted mammary epithelial cell proliferation, casein, and triglyceride synthesis. In this process, ORP5 also promotes the lysosomal localisation of mTOR and increases the level of mTOR phosphorylation [144]. The mTOR signalling pathway has been shown to be critical in milk synthesis [145]. The process described is shown in Figure 5.

Myristic acid (MA) is a straight-chain saturated fatty acid first found in nutmeg, the natural source of which is mainly palm trees and a variety of tropical fruits [146]. In contrast to palmitic and stearic acids, myristic acid is particularly present in milk fat [147]. It acts as a precursor substance for fatty acid synthesis and is converted into myristoyl coenzyme A by carboxylases. Myristic acid can act as a stimulant during lipid synthesis and promotes triglyceride synthesis by enhancing the expressions of ACACA and FASN [148]. Hu and colleagues treated dairy cow mammary epithelial cells with different concentrations of myristic acid solutions and found that triglycerides became abundant and larger lipid droplets appeared. In addition, the levels of ubiquitin proteins and ubiquitination signalling pathways were significantly increased with treatment using myristic acid, but it did not affect proteasome activity [149]. In Hu’s study, CD36 and ADFP protein levels were also found to be significantly upregulated, suggesting enhanced fatty acid transport and lipid droplet formation (Figure 5). Although the exact regulatory mechanism has not yet been clarified, this provides a basis for our subsequent studies on the ubiquitination or de-ubiquitination of myristic acid in the control of milk fat production.

In fact, plant extracts, such as carotenoids, anthocyanins, and allicin, can also be used as feed additives for dairy cows. They show improved feed utilisation, oxidative stress, and inflammation in mammary epithelial cells, as well as an increase in milk yield and milk fat percentage during lactation [150]. It has been demonstrated that they can also affect ubiquitination modifications, such as in human hepatocytes, where allicin reduces the ubiquitination of Sestrin2, an important family of stress-inducible proteins, contributing to the homeostasis of the intracellular environment [151]. Anthocyanins indirectly reduce ubiquitination of Nrf2 protein by decreasing Keap1 protein, and more Nrf2 enters the nucleus to initiate downstream target genes to antagonise oxidative stress in mouse small intestinal epithelial cells [152].

The above study reveals that it is of research interest to include these plant extracts that can be used as feed additives in the study of ubiquitination and de-ubiquitination in regulating milk fat synthesis in dairy cows. At the same time, this work may provide some new directions for dairy cattle feeding, with a view to improving the milk fat percentage. Studying the effects of food and plant extracts on milk synthesis and elucidating the mechanisms are important for enhancing nutritional management practices in dairy cows and dairy product quality.

## 5. Ubiquitination and De-Ubiquitination Modification Linked to Cows’ Breast Health

The development of mastitis in dairy cows can reduce milk production and severely affect milk fat levels and the flavour of dairy products [153]. Especially susceptible to high temperatures and humidity, heat shock proteins (Hsps) are upregulated in the mastitis response [154]. Heat shock proteins are a group of stress-induced proteins involved in protein folding and maturation and are highly conserved in mammals [155]. In addition, it was found that heat shock proteins HSP27 and Hsp90 can also activate the NF-κB signalling pathway by directing ubiquitination degradation following phosphorylation of the protein I-κBα (NF-κB inhibitor alpha), which in turn plays an important role in the inflammatory response [156]. It has been shown that heat shock proteins themselves can also be directly subjected to ubiquitination and de-ubiquitination modifications, such as the ubiquitination of Hsp90, which inactivates its chaperone function and destabilises excess client proteins [157]. Hsp27 induces the ubiquitination-like SUMOylation of Hspb8 to promote Hspb8 protein stability, which in turn supports breast cancer cell proliferation and metastasis [158]. Furthermore, it was found that the de-ubiquitinating enzyme USP40 maintains endothelial cell integrity in mice by targeting the heat shock protein Hsp90 to mitigate inflammatory responses [159]. It is reasonable to imagine that ubiquitination and de-ubiquitination could be involved in cow mammary gland health to ensure normal milk fat synthesis, but this is yet to be verified and explored by future dairy workers and molecular breeding researchers.

## 6. Concluding Remarks and Outlook for the Future

The dynamic equilibrium of ubiquitination and de-ubiquitination plays an important role in fatty acid transport, de novo synthesis, desaturation, and lipid droplet formation in dairy cows. Abnormalities in the ubiquitination–proteasome and lysosomal pathways can lead to alterations in the ubiquitination levels of the mammary epithelium of dairy cows, directly affecting milk fat synthesis. Natural plant extracts used as feed additives in dairy cattle feed even regulate the activity of enzymes related to milk fat synthesis via the ubiquitination pathway. Beyond this, it is interesting that ubiquitination and de-ubiquitination seem to be involved in breast health. Indeed, ubiquitination and de-ubiquitination, although hot research areas, have been little studied in dairy cows. A great deal of research is still needed on how ubiquitination and de-ubiquitination regulate the degradation and biological activity of lactolipid-associated proteins, the type of ubiquitination involved in the modification process, and the types and localisation of ubiquitinating and de-ubiquitinating enzymes. However, an in-depth study of the biological functions of the ubiquitination and de-ubiquitination pathways during milk fat production in dairy cows and their specific modes of action is expected to reveal a new ubiquitin-centred mechanism for regulating milk fat synthesis and to provide a reference for the study of ubiquitination and de-ubiquitination in other production traits of dairy cows.
